# A Randomised, Double-Blind, Placebo-Controlled Trial with Vitamin D3 in MS: Subgroup Analysis of Patients with Baseline Disease Activity Despite Interferon Treatment

**DOI:** 10.1155/2012/802796

**Published:** 2012-08-05

**Authors:** J. Åivo, B.-M. Lindsröm, M. Soilu-Hänninen

**Affiliations:** ^1^Department of Neurology, Turku University Hospital and University of Turku, Kiinamyllynkatu 4-8, 20520 Turku, Finland; ^2^4Pharma Ltd., 20520 Turku, Finland

## Abstract

We present a subgroup analysis of the first double-blind, placebo-controlled, randomised trial with vitamin D3 in MS. In the overall study population, there were 34 patients in the vitamin D arm and 32 patients in the placebo arm. All the patients were using interferon-**β**-1b (IFNB) therapy. The subgroup consisted of 15 patients in the vitamin D arm and 15 patients in the placebo arm, who had either at least one relapse during the year preceding the study or enhancing T1 lesions at the baseline MRI scan. We measured the total number of MRI T1 enhancing lesions, the number of new/enlarging T2 lesions and T2 lesion volume (BOD) (mm^3^), EDSS (Expanded Disability Status Scale), annual relapse Rate (ARR), timed 25-foot walk (T25FW), and timed 10-foot tandem walk (TT10W) at baseline and at 12 months in the vitamin D-treated and in the placebo-treated patients. There was a statistically significant reduction in the number of T1 enhancing lesions, a smaller T2 lesion volume growth and less new/enlarging T2 brain MRI lesions in the vitamin D3-treated than in the placebo-treated subgroup patients. The MRI results were slightly more pronounced in the subgroup than in the overall study population.

## 1. Introduction

In a previously published double-blind, placebo-controlled trial in MS patients using IFNB therapy, we found that 20,000 IU of vitamin D3 once weekly increased the mean circulating 25-hydroxyvitamin D (25(OH)D) from 54 nmol/L to 110 nmol/L over one year [1]. This increase resulted in significantly fewer gadolinium enhancing lesions on brain MRI and strong trends toward lower T2 lesion burden, reduced EDSS, and improved timed 10-foot tandem walk scores compared to controls, whose mean circulating 25(OH)D was unchanged [1]. The was no hypercalcaemia or other treatment-related adverse events [1]. Several other studies in patients with MS have suggested a link between vitamin D status and disease activity. In children with a first demyelinating event (FDE), each 10 nmol/L decrease in 25(OH)D has been correlated with a conversion to definite MS [2]. Lower serum 25(OH)D values have been associated with a higher rate of MS relapses [3–5]. Most recently, researchers correlated each 10 nmol/L increase in 25(OH)D with up to 12% reduction in relapse rate in adults with MS [6], and each 25 nmol/L increase in 25(OH)D with a 34% decrease in relapse rate in pediatric-onset MS [7]. Other researchers have correlated greater early sun exposure and vitamin D intake with a reduced risk of progression to severe disability in veterans with MS [8], and lower 24,25-dihydroxyvitamin D (24,25(OH)_2_D) with higher Expanded Disability Scale (EDSS) and higher 25(OH)D to 24,25(OH)_2_D ratio with lower brain parenchymal fraction in patients with MS [9]. A one-year randomized controlled vitamin D3 dose escalation study (mean intake ~14,000 IU/day), although not powered to detect clinical outcomes, nevertheless found that the proportion of patients experiencing relapses was lower in the vitamin D3-supplemented group than in the control group [10].

MS patients with higher clinical disease activity tend to benefit from immune modulating therapies more than clinically less active patients [11, 12]. In this paper, we have performed a subgroup analysis of the Finnish Vitamin D Study to assess whether patients with relapses during the year preceding the study or Gadolinium enhancing T1 lesions at the baseline MRI scans in spite of IFNB therapy benefited of the vitamin D3 add-on therapy in comparison with add-on placebo.

## 2. Materials and Methods

### 2.1. Patients

**Figure 1:**
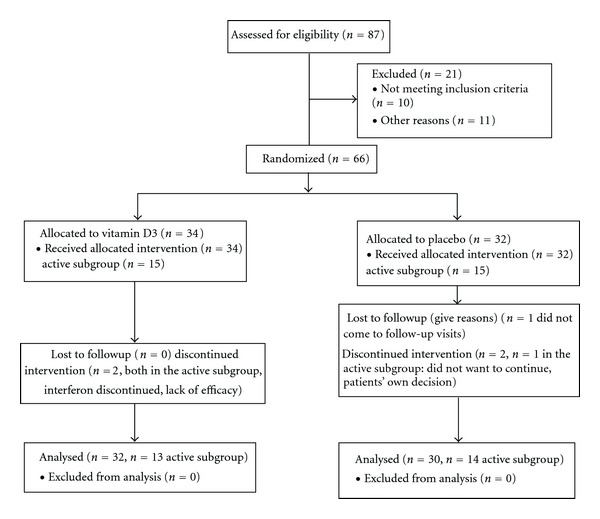
Study flow chart.

We performed a preplanned subgroup analysis of the Finnish Vitamin D Study, a double-blinded, randomised, parallel group, one-year trial with add-on vitamin D3 in relapsing-remitting MS patients receiving subcutaneous IFNB-1*β* therapy. The trial is registered in EudraCT (number 2007-001958-99) and ClinicalTrials.gov (NCTO1339676). The subgroup consisted of patients who had either Gadolinium (Gd)-enhancing T1 lesions on brain MRI at the study baseline or at least one relapse within the year preceding the study baseline. At the study baseline, there were 34 patients in the vitamin D group and 32 patients in the placebo group altogether and 15 patients in the vitamin D group and 15 patients in the placebo group in the subgroup of active patients. A total of 32 patients in the vitamin D group and 30 patients in the placebo group completed the study. One patient in the subgroup of active patients dropped out in the placebo arm and two in the vitamin D arm such that 13 patients receiving vitamin D and 14 patients receiving placebo in the active subgroup were included in the analysis presented in this paper. Flow of the patients in the study is shown in Figure 1.

The study protocol of the Finish Vitamin D Study was approved by the joint ethics committee of the Turku University and the Turku University Hospital as well as the the National Agency for Medicines (Finland). The study was undertaken in accordance with the declaration of Helsinki and The European Medicines Agency Note for Guidance on Good Clinical Practise. Patients were recruited from the outpatient policlinics of Turku, Helsinki, Tampere, Oulu and Kuopio University Hospitals and Central Hospitals of Central Finland and Ostrobotnia.

Inclusion criteria were age 18 to 55 years; relapsing-remitting MS according to the McDonald criteria; INFB therapy for at least one month; EDSS < 5; no neutralizing antibodies to INFB; appropriate contraceptive methods; signed written informed consent. In this subgroup analysis, we included patients with active disease: at least one relapse duringthe year preceding the study and/or MRI activity defined as presence of Gd-enhancing lesions on brain MRI at the study baseline.

Exclusion criteria were serum calcium >2,6 mmol/L; serum 25(OH)D >85 nmol/L; primary hyperparathyroidism; any condition predisposing to hypercalcaemia; pregnancy or unwillingness to use contraception; use of other immunomodulatory therapy than INFB-1*β* (Betaferon); known allergy to cholecalciferol or peanuts; sarcoidosis; renal insufficiency; significant hypertension (BP > 180/110); hyperthyroidism or hypothyroidism; a history of kidney stones during the previous five years; cardiac insufficiency or significant dysrhythmia; major depression; inability to perform serial MRI scans; alcohol or drug abuse.

### 2.2. Randomization and Masking

 Patients were randomized 1 : 1 to treatment with either cholecalciferol or placebo. A separate randomisation was done for each centerusing randomly permuted blocks. The randomization was performed at 4Pharma Ltd using SAS for Windows software version 8.2. All study personnel and participants were blinded to the treatment code until the data base was locked. 

### 2.3. Study Product

20 mg of cholecalciferol (Dekristol) corresponding to 20000 IU (500 *μ*g) of vitamin D3, administered as a capsule once a week, or identically appearing placebo capsules (Swiss-Caps, Switzerland) were used. A private company (Joutsen apteekki, Turku, Finland) organized the importing, packaging, and labelling procedure of the study product. Patients were given a bottle of 26 capsules of study medication and were instructed by the investigator in the weekly administration of the study medication. At month 6, a new bottle of 26 capsules of study medication was given to patients. Adherence was evaluated by capsule counting. 

### 2.4. Procedures

There were 6 study visits during 12 months. At the screening visit, concurrent illnesses and medications were recorded. Physical examination, EDSS, height, weight and heart rate, blood pressure and electrocardiogram, the timed 10-foot tandem test (TTW10) and timed 25-foot walk test (T25FW) were performed. A mean of two attempts of both walk tests was used in the analyses. MRI was done within 2 weeks before or at the randomisation visit. 25(OH)D, as well as MxA to indirectly assess neutralizing antibodies to IFNB, was measured at baseline and at 6 and 12 months. Patients with a lack of MxA response at the screening, indicating presence of neutralizing antibodies to IFNB, were excluded from the study. EDSS and timed walk test were monitoredat baseline and at 12 months. Patients contacted the study centres for unscheduled visits within 7 days of relapse onset. At the unscheduled visits, EDSS was performed and the investigator defined whether a relapse had occurred. At the judgement of the treating neurologist, relapses were treated with methylprednisolone 1 g daily for three days. 

A commercially available assay, 25-hydroxy vitamin D ^125^I RIA kit (DiaSorin Catalogue number 68100E, Stillwater, MN, USA) was used for 25(OH)D measurement as described previously (3,4). Two quality control samples were included in each assay series, and the specimens and controls were assayed in duplicate. The sensitivity of this assay in 4.0 nmol/L and the interassay coefficient of variation in <10%.

### 2.5. MRI Acquisition and Analysis

A standardized MRI study was performed in each centre using a 1.5 T scanner. MRI was done within 2 weeks before or at the randomisation visit and within 2 weeks before or at the final visit. A dummy run MRI was performed before a site was accepted into the trial. Central analyses were performed at the Neuroimaging Research Unit, Vita-Salute San Raffaele University in Milan, Italy and included quantification of the total number of gadolinium enhancing T1 lesions and the number of new/enlarging T2/PD lesions and T2 lesion volume (BOD) (mm^3^). 

### 2.6. Statistical Analysis

SAS V.9.2 was used for all analyses. Nonparametric rank analysis of covariance was used in analysing MRI T2 BOD and EDSS at 12 months with baseline values as covariates, controlling for centre. ANCOVA was used to analyse the change in TTW10 or T25FW (after log transformation) with TTW10 and T25FW at baseline and centre as covariates. Numbers of Gd-enhancing lesions on T1 and new/enlarging lesions of T2 scans were analysed using a generalised mixed model based on Poisson distribution. A *P* value <0.05 was considered statistically significant.

## 3. Results

The results of the intention to treat analysis of all patients have been published in February 2012 [1]. In this paper, we present the results of the subgroup of patients who had either MRI activity or clinical disease activity at the study baseline. Clinical and laboratory characteristics of the subgroup are shown in Table 1. Serum 25(OH)D levels rose from a mean of 55 nmol/L (range 35–82) to 115 nmol/L (range 78–163) in the vitamin D-treated patients after 12 months of treatment and remained unchanged in the placebo group (mean of 50 nmol/L [range 24–81] at baseline and 48 nmol/L [range 30–68] at 12 months). Lack of MxA at 12 months was detected in two patients in both treatment arms (none at baseline because lack of MxA was an exclusion criterion). MRI characteristics of the subgroup at baseline and at 12 months are shown in Table 2. The first patient was screened in March 2008 and randomised in April 2008. The last patient was randomised in May 2010 and completed the study in May 2011. The data base was locked inAugust 2011.

### 3.1. MRI T2BOD

MRI T2 BOD increased more in the placebo subgroup (median change 570 mm^3^) compared to the vitamin D treated subgroup patients (median change 104 mm^3^) but the difference between the treatment groups was not statistically significant (*P* = 0.105, Table 2).

### 3.2. MRI Activity, Number of Gadolinium-Enhancing T1 Lesions, and Number of New/Enlarging T2 Lesions

The number of gadolinium enhancing lesions on T1 decreased statistically significantly in both groups (*P* = 0.018) but statistically significantly more in the vitamin D-treated group (*P* = 0.027, Table 2). The percentage of patients with MRI activity at 12 months was lower in the vitamin D-treated patients but the difference did not quite meet statistical significance (*P* = 0.08, Table 2). The number of new or enlarging lesions on T2/PD weighted scans at 12 months was greater in the placebo group, but the difference was not statistically significant either (*P* = 0.132, Table 2).

### 3.3. Time to First Relapse, ARR, EDSS, TTW10, and T25FW

There was not a statistically significant difference between the groups in time to first relapse during the study in patients receiving vitamin D in addition to IFNB in comparison with patients receiving add-on placebo (*P* = 0.794, hazard ratio 0.84, 95% CI 0.23 to 3.1). Adjusting to EDSS at baseline affected the hazard ratio in favour of vitamin D, but there was still no difference between the groups (hazard ratio 0.74, 95% CI 0.20 to 2.80). The baseline ARR in the vitamin D-treated active subgroup patients was 0.61 and in the placebo-treated subgroup patients 0.83. At 12 months, the mean ARR in the vitamin D-treated patients was 0.33 and in the placebo-treated patients 0.47. The difference was in favour of vitamin D but not statistically significant. There was no change in median EDSS in neither treatment group (*P* = 0.274, Table 3). There was no statistically significant difference between the treatment groups neither in T25FW nor in TTW10 (Table 3).

## 4. Discussion

The Finnish Vitamin D Study was the first randomized, double-blind, placebo-controlled trial examining the effects of vitamin D3 in MS. No significant difference was seen in the clinical parameters, but vitamin D3 add-on treatment to INFB significantly reduced the number of Gd-enhancing lesions on brain MRI [1]. In the subgroup analysis of active patients presented in this paper, the effects of vitamin D3 on the number of Gd-enhancing lesions were statistically significant as well although the number of patients in the subgroup was only 15 in the vitamin D group and 15 in the placebo. In the overall study population, there were 34 patients in the vitamin D arm and 32 patients in the placebo arm. 

The effects on MRI activity and MRI T2 BOD were more pronounced in the subgroup than in overall study population, but did not reach statistical significance. In the subgroup, 23% of vitamin D3-treated and 57% of placebo-treated patients had MRI activity (new or enlarging T2 lesions or Gd-enhancing lesions) at 12 months (*P* = 0.08). In the overall study population, there was MRI activity in 25% of the vitamin D3-treated patients and 37% of the placebo patients at 12 months (*P* = 0.322). The mean number of new or enlarging T2 lesions was 0.6 in the vitamin D3 treated patients and 1.9 in the placebo treated patients (*P* = 0.132) in the subgroup and 0.5 and 1.1, respectively, in the overall study population (*P* = 0.286). In the subgroup, MRI T2 BOD increased a median of 570 mm^3^ in the placebo patients and 104 mm^3^ in the vitamin D3 treated patients. In the overall study population, MRI T2 BOD increased a median of 287 mm^3^ in the placebo group compared to 83 mm^3^ in the vitamin D3 group. The differences in MRI T2 BOD between the groups were not statistically significant neither in all of the patients nor in the active subgroup, although the mean of 466 mm^3^ smaller lesion volume growth in the vitamin D-treated subgroup appears clinically significant. The sample size was calculated such that with 40 patients in each treatment arm a difference of 1000 mm^3^ in BOD would have been detected with a power of 80%.

MS patients with high disease activity seem to benefit more from disease-modifying therapies (DMTs) than patients with less disease activity. Patients who start the treatment later in the disease course do not gain the same benefits as those who begin treatment early in the course of multiple sclerosis [13]. DMT has also been suggested to have greater benefits in younger patients because of the higher rate of relapsing activity reported in younger patients with MS [14]. Natalizumab has been shown to have the greatest effect in patients with more disease activity [15, 16]. In the post hoc analysis of AFFIRM and SENTINEL, it was shown that natalizumab is effective in reducing the risk of disability progression particularly in patients with highly active disease [11]. In the FREEDOMS study, the efficacy of fingolimod on ARR was more pronounced in patients with high baseline activity [12]. In cladribine-treated patients, a greater effect on disease activity was described in patient subgroups with high relapse or lesion activity at the baseline. The most favourable responders were those with a shorter disease duration or those with one or more T1 Gd-enhancing lesion at the baseline [17]. In 15 patients with monthly MRI scans a trend was visible that INFB responders had a higher total number of Gd-enhancing lesions during the pretreatment period [18]. Active lesions, either Gd-enhancing T1 lesions or new or enlarging lesions on T2 scans, are good predictors of relapses at population level. A strong correlation between the effect of therapy on active lesions and its effect on the relapse rate has been identified, suggesting that MRI could serve as a surrogate marker for relapses in MS [19]. 

In the overall study population of Finnish Vitamin D Study, clinical outcomes appeared to favor the high-dose vitamin D group. In the subgroup of active patients, there was no difference between the vitamin D and the placebo groups. Due to the small sample size, the study was not powered to address clinical outcomes. At the same time with our study, a Norwegian randomized, placebo-controlled trial in 68 RRMS patients with the same vitamin D3 compound as we used in our trial was published [20]. The study found no difference between the treatment groups, and none of the clinical parameters were in favor of the vitamin D group. There was no MRI analysis in the study by Kampman and coworkers and the patients were clinically less active (mean baseline ARR 0.11 in comparison with 0.51 in our study [1] and 0.72 in the subgroup). Mean serum level of 25(OH)D in the placebo group at the study end was slightly higher in the Norwegian trial than in ours (61.8 nmol/L versus 50 nmol/L), since in Norway the placebo group patients were allowed to continue the vitamin D supplements that they were using at baseline. The baseline 25(OH)D levels in the placebo group were almost identical in Norway and in Finland (57 and 56 nmol/L, resp.). Moreover, there were differences between the trials in the immunomodulatory treatments that the patients were using (30/68 patients were using interferons in the Norwegian trial, whereas all the patients were using IFNB1b in our trial). It is possible that IFNB and vitamin D3 have a synergistic effect in MS. 

## 5. Conclusions

We performed a subgroup analysis of the Finnish Vitamin D Study. All patients were using subcutaneous IFNB1b 250 *μ*g three times a week. A total of 15 placebo-treated and 15 vitamin D3-treated patients with either a relapse during the year preceding the study baseline or Gd-enhancing T1 lesions in the baseline brain MRI were included in the subgroup analysis. There was a statistically significant reduction in the number of T1-enhancing lesions and smaller lesion volume growth and less MRI disease activity in the vitamin D3-treated than in the placebo treated subgroup patients after 12 months of vitamin D3 (500 *μ*g per week)) or identically appearing placebo add-on therapy. The MRI results were slightly more pronounced in the active subgroup than in the overall study population. The sample size was too small to assess the impact of vitamin D on clinical measures of disease activity, progression, and function. 

## Figures and Tables

**Table 1:** Patient characteristics and serum 25(OH)D levels in the vitamin D-and placebo-treated active subgroup patients.

Characteristics	Vitamin D	Placebo
Number of patients	15	15
Gender (F/M)	9/6	9/6
Age (median, range)	37 (25–53)	32 (22–47)
BMI (median, range)	23.8 (19.7–31.2)	25.5 (19.3–32.3)
EDSS (median, range)	2 (0–3.5)	2 (0–4)
Disease duration (years, median, range)	3 (0.6–15.2)	1.5 (0.3–4.7)
ARR (mean, SD)	0.67 (0.38)	0.83 (0.37)
Duration of interferon therapy (months, median, range)	23 (4–82)	10 (2–53)
Vitamin D intake (*μ*g, mean, SD)	2.04 (1.49)	2.71 (1.41)
Serum 25(OH)D at baseline (nmol/L, mean, range)	55 (35–82)	50 (24–81)
Serum 25(OH)D at 12 months (nmol/L, mean, range)	115 (78–163)	48 (30–68)
Calcium intake (mg, mean, SD)	1240 (357)	1357 (383)

BMI: body mass index; EDSS: Expanded Disability Status Scale; ARR: annual relapse rate.

**Table 2:** MRI results in the vitamin D-and placebo-treated active subgroup patients.

			Vitamin D		Placebo	*P* value
T2 BOD (mm^3^), median (SE)	Baseline	*n* = 15	4391 (2305)	*n* = 15	9930 (2375)	0.105
	Change from baseline	*n* = 13	104 (240)	*n* = 14	570 (3259)	
Number of T1-enhancing lesions, mean (SD)	Baseline	*n* = 15	1.5 (3.6)	*n* = 15	1.5 (2.9)	
	Month 12	*n* = 13	0.1 (0.3)	*n* = 14	1.5 (5.0)	0.027^∗^
Patients with T1-enhancing lesions (*n*,%)	Baseline	*n* = 15	6 (40%)	*n* = 15	6 (40%)	
	Month 12	*n* = 13	1 (8%)	*n* = 14	3 (21%)	
Number of new or enlarging T2/PD lesions, median (SD)	Month 12	*n* = 13	0.61 (1.26)	*n* = 14	1.85 (2.44)	0.132
Patients with new or enlarging lesions on T2/PD, mean (SD)	Month 12	*n* = 13	3 (23%)	*n* = 13	8 (57%)	NS
MRI activity (*n*,%)	Baseline	*n* = 15	6 (40%)	*n* = 15	6 (40%)	0.080
	Month 12	*n* = 13	3 (23%)	*n* = 14	8 (57%)	

BOD: burden of disease; MRI activity: Gadolinium-enhancing T1 lesions or new/enlarging T2 lesions; *n*: number of patients. ^∗^Indicates statistically significant. NS: not significant.

**Table 3:** Clinical outcomes in vitamin D- and placebo-treated active subgroup patients.

	Vitamin D		Placebo		*P* value
	Baseline	Month 12	Baseline	Month 12
EDSS mean (SD) change	2.2 (0.9)	1.9 (1.1)	1.9 (1.2)	2.0 (1.5)	0.274
		−0.3 (0.6)		−0.1 (0.7)	
ARR, mean (SD)	0.67 (0.38)	0.33 (0.62)	0.83 (0.37)	0.47 (0.74)	NS
TTW10, mean (SD) change (seconds)	12.17 (6.27)	10.36 (2.70)	10.35 (7.39)	12.10 (13.5)	0.820
		−2.32 (4.62)		1.16 (7.65)	
T25FW, mean (SD) change (seconds)	7.26 (7.8)	5.27 (1.4)	4.54 (0.94)	5.08 (1.14)	0.860
		−1.85 (8.1)		0.44 (0.92)	

EDSS: Expanded Disability Status Scale; ARR: annual relapse rate; TTW10: timed 10-foot tandem walk; T25FW: timed 25-foot walk.
